# An Occupancy Mapping Method Based on K-Nearest Neighbours

**DOI:** 10.3390/s22010139

**Published:** 2021-12-26

**Authors:** Yu Miao, Alan Hunter, Ioannis Georgilas

**Affiliations:** Department of Mechanical Engineering, University of Bath, Bath BA2 7AY, UK; A.J.Hunter@bath.ac.uk (A.H.); I.Georgilas@bath.ac.uk (I.G.)

**Keywords:** mapping, SLAM, data sets for SLAM

## Abstract

OctoMap is an efficient probabilistic mapping framework to build occupancy maps from point clouds, representing 3D environments with cubic nodes in the octree. However, the map update policy in OctoMap has limitations. All the nodes containing points will be assigned with the same probability regardless of the points being noise, and the probability of one such node can only be increased with a single measurement. In addition, potentially occupied nodes with points inside but traversed by rays cast from the sensor to endpoints will be marked as free. To overcome these limitations in OctoMap, the current work presents a mapping method using the context of neighbouring points to update nodes containing points, with occupancy information of a point represented by the average distance from a point to its k-Nearest Neighbours. A relationship between the distance and the change in probability is defined with the Cumulative Density Function of average distances, potentially decreasing the probability of a node despite points being present inside. Experiments are conducted on 20 data sets to compare the proposed method with OctoMap. Results show that our method can achieve up to 10% improvement over the optimal performance of OctoMap.

## 1. Introduction

As an environment perception approach for autonomous mobile robots, occupancy mapping is widely used in path planning [1,2], navigation [3,4] and autonomous driving [5,6]. In most applications, occupancy maps are generated from point clouds. Sensors such as LIDAR [7,8] can produce high-quality point clouds to represent the 3D world. However, such sensors are normally expensive, which has restricted their application. Currently, point clouds are becoming increasingly popular in the research community as cheaper solutions become available. An RGB-D camera [9,10] can simultaneously produce both colour and depth images, the latter of which can be used for point cloud reconstruction. Similarly, point clouds can be reconstructed from a stereo camera [11,12] with the disparity map derived by left and right images.

To build occupancy maps from point clouds, OctoMap is a popular mapping algorithm. OctoMap is octree-structure-based and uses probabilistic occupancy estimation to update maps [13]. An octree is a hierarchical data structure in which each node is recursively divided into eight children [14,15]. In OctoMap, the volumes of a 3D space are represented by the cubic nodes in the octree and the probability of each node is updated with the measured endpoints in point clouds. It is assumed that the endpoints correspond to obstacle surfaces and there are no objects between sensor origin and endpoints. A ray-casting operation from the senor to endpoints will be performed to determine which nodes should be updated. A node will be updated accordingly with the probabilities in the inverse sensor model if the node is traversed by the beam cast from the sensor or the beam is reflected in the node.

However, the map update policy in OctoMap has limitations when dealing with nodes containing points. Normally, point clouds are either generated by sensors directly or by implementing algorithms on the original data from sensors. Due to sensor noise and the limitations of point cloud generation algorithms, a point cloud normally contains both points on the external surfaces of objects and noise points. One one hand, in OctoMap, the nodes containing points are updated with the same probability regardless of points being noise. On the other hand, with a single frame of point cloud, the probability of a node with points inside can be increased but is never allowed to be decreased. In addition, potentially occupied nodes containing points but traversed by rays will be marked as free. The limitations of the update policy in OctoMap have a negative impact on the mapping performance. Since OctoMap classifies the nodes in a map into occupied and free ones, it can be treated as a binary classifier. The classification performance can be described by a confusion matrix [16] in which elements are categorised into true positives (TP), false positives (FP), true negatives (TN) and false negatives (FN). The occupied nodes related to noise points will be classified as FPs and nodes with points inside but traversed by rays are likely to introduce FNs.

Considering occupancy mapping using point clouds has a wide range of applications in multiple fields, and the map update policy can be improved to enhance the mapping performance. Point cloud filters such as those in the Point Cloud Library (PCL) [7] can be used for denoising. Instead of building a map with the original point clouds, the quality of the map can be improved using the filtered point clouds. However, the details in environmental features may not be preserved when the outliers or clusters in point clouds are removed by filters. To refine occupancy maps, Recurrent-OctoMap is proposed in [17], with each cell in OctoMap modelled as a Recurrent Neural Network (RNN). The learning approach is used to fuse semantic features. In [18], learning-aided 3D occupancy mapping is introduced to deal with sparse and noisy range sensor data. The occupancy states of the unobserved regions can be predicted to build dense occupancy maps. Previous studies reported in the literature also focused on the improvement of the efficiency of occupancy mapping. In [19], a computationally efficient probabilistic map update policy utilising the sparse nature of the environment is proposed. The efficiency of the OctoMap framework can also be improved by the Fast Line Rasterisation Algorithm [20].

The purpose of this work is to overcome the limitations in OctoMap by representing occupancy information with the average distance from a point to its k-Nearest Neighbours (k-NN). The main contributions of this paper are:

- a k-NN method for occupancy mapping using the context of neighbouring points to update nodes containing points;
- definition of the relationship between the average distance and the change in occupancy probability, potentially decreasing the probability of a node despite the points being present in the node;
- the proposed k-NN method is verified by the point clouds derived by the StereoSGBM algorithm [21] implemented on the images produced from a stereo camera, and can be potentially extended to other point-cloud-based mapping systems.

Both OctoMap and the k-NN-based mapping approach are governed by several parameters, the choice of which affects the quality of the final map. To compare the k-NN method with OctoMap, it is reasonable to compare their optimal mapping performance, which can normally be achieved by tuning parameters. A two-step methodology is introduced in [22] to identify optimal parameter sets to improve occupancy mapping performance by first reducing parameters of lower impact using Neighbourhood Component Analysis (NCA) [23] and then optimising the residual most significant ones with grid search. In [22], OctoMap is implemented on 20 data sets collected in two environments to show the effectiveness of the parameter reduction and optimisation method. In this work, the data sets introduced in [22] are used to demonstrate the benefits of the novel k-NN method over OctoMap by comparing their optimal performance derived by the two-step principled methodology as per [22].

This paper is organised as follows. We first introduce the background of OctoMap in Section 2. Then, the k-NN-based inverse sensor model is presented in Section 3, using the relationship between the average distance from a point to its k-NN and occupancy probability. The relationship is defined with the Cumulative Density Function (CDF) of average distances and the choice of the distribution is explained. The map update procedure using the proposed model is given as well. In addition, considerations of parameter space are specified, followed by the introduction of parameter reduction based on NCA and optimisation using grid search. In Section 4, the details of the experiments are given. The results of average distances fitted by different distributions, k-NN parameter reduction and the comparison of the k-NN method and OctoMap are also presented. Finally, the key findings of the CDF of the average distance nonsensitive to distributions, lower impact of parameter *k* compared to other k-NN parameters and the improvement by the k-NN method up to 10% over the optimal performance of OctoMap are discussed.

## 2. Background

We compare the k-NN method with OctoMap, the details of which have been explained in [13]. A brief summary of the update policy of OctoMap is introduced here.

In OctoMap, sensor readings are integrated with the occupancy grid mapping method introduced in [24]. Let $z_{1:t}$ denote sensor measurements up to time *t*. The probability of a node $m_{i}$ can be written as:

(1) $$p\left(m_{i}|z_{1:t}\right)={\left[1+\frac{1-p(m_{i}|z_{t})}{p(m_{i}|z_{t})}\frac{1-p(m_{i}|z_{1:t-1})}{p(m_{i}|z_{1:t-1})}\frac{p(m_{i})}{1-p(m_{i})}\right]}^{-1}\;,$$

where $p(m_{i}|z_{t})$ is the probability given measurement $z_{t}$, and $p(m_{i})$ denotes the prior probability and is commonly set to 0.5.

The log-odds notation

(2) $$l\left(x\right)=\ln\left(\frac{x}{1-x}\right)$$

can be used to simplify (1) as:

(3) $$l\left(m_{i}|z_{1:t}\right)=l\left(m_{i}|z_{1:t-1}\right)+l\left(m_{i}|z_{t}\right)\;,$$

where $l(m_{i}|z_{t})$ is the inverse sensor model. As introduced in Section 1, the nodes that need to be updated are determined by a ray-casting operation from sensor origin to endpoints. The probabilities corresponding to nodes containing points and traversed by the beam are given in the inverse sensor model:

(4) $$l\left(m_{i}|z_{t}\right)=\{\begin{array}{cc} l_{occ} & \mathrm{if}\;\mathrm{containing}\;\mathrm{points}\\ l_{free} & \mathrm{if}\;\mathrm{traversed}\;\mathrm{by}\;\mathrm{the}\;\mathrm{beam} \end{array}\;,$$

where $l_{occ}$ and $l_{free}$ are the respective log-odds values to update occupied and free cells. The log-odds value of the probability is limited by the clamping update policy proposed in [25]:

(5) $$l\left(m_{i}|z_{t}\right)=\max\left(\min\left(l\left(m_{i}|z_{1:t-1}\right)+l\left(m_{i}|z_{t}\right),l_{\max}\right),l_{\min}\right)\;,$$

where $l_{\max}$ and $l_{\min}$ are the upper and lower bounds on the probability in log-odds notation, respectively.

## 3. Method

In this work, the context of neighbouring points is used to update occupancy maps. We first define the relationship between the average distance from a point to its k-NN and the occupancy probability. An inverse sensor model is proposed based on the relationship. Using the proposed model, the mapping algorithm is governed by several parameters. These parameters are reduced by NCA and optimised with grid parameter space to achieve the best mapping performance.

### 3.1. K-NN-Based Inverse Sensor Model

In a point cloud, a point is likely to be noise if it is isolated from the points nearby. Based on this assumption, the average distance from a point to its k-NN can be used to represent the occupancy information of this point. A point should be assigned with a higher probability if it has a smaller average distance, and vice versa. We have defined a relationship between the average distance and the change in probability in [26]. In this paper, we make minor changes and redefine the relationship. Let f(x) denote the distribution of the average distance. Then, the probability representing the occupancy information of a point can be denoted as:

(6) $$p\left(s\right)=p_{u}-\frac{\int_{-\infty }^{s}f\left(x\right)dx}{\int_{-\infty }^{\infty }f\left(x\right)dx}\left(p_{u}-p_{l}\right)≔p_{u}-F\left(s\right)\left(p_{u}-p_{l}\right)\;,$$

where *s* is the average distance from a point to its k-NN, $p_{u}$ and $p_{l}$ are the upper and lower bounds on the probability, and F(s) is the CDF of the average distance and $F\left(s\right)=\int_{-\infty }^{s}f\left(x\right)dx$.

Based on the relationship, we define the inverse sensor model as:

(7) $$l\left(m_{i}|z_{t}\right)=\{\begin{array}{ll} \sum_{j}l\left[p(s_{j})\right] & \mathrm{if}\;\mathrm{the}\;j\mathrm{th}\;\mathrm{point}\;\mathrm{in}\;\mathrm{node}\;m_{i}\\ l_{in} & \mathrm{if}\;\mathrm{traversed}\;\mathrm{by}\;\mathrm{rays}\;\mathrm{and}\;\mathrm{endpoints}\;\mathrm{within}\;\mathrm{range}\;s_{c}\\ l_{out} & \mathrm{if}\;\mathrm{traversed}\;\mathrm{by}\;\mathrm{rays}\;\mathrm{and}\;\mathrm{endpoints}\;\mathrm{outside}\;\mathrm{range}\;s_{c} \end{array}\;,$$

where $s_{c}$ is the maximum range for how long individual beams are inserted, and $l_{in}$ and $l_{out}$ are the respective log-odds values assigned to the nodes traversed by rays cast from the sensor to the points whose distances to the sensor are within and outside the range $s_{c}$. If a node satisfies both the requirements of $l_{in}$ and $l_{out}$, it will be updated with $l_{in}$ only. Then, we can use (3) and (5) to update an occupancy map.

With the above inverse sensor model and the OctoMap parameters introduced in [22], the parameters of k-NN mapping are as follows.

- $p_{\max}$ is the upper clamping threshold, which is the upper bound on the probability.
- $p_{t}$ is the threshold. A node will be marked as occupied when the threshold is reached.
- $p_{m}$ is the probability of a “miss”. A node will be updated with $p_{m}$ if it is traversed by rays and corresponding endpoints are within range $s_{c}$.
- $p_{m}^{\prime }$ is the probability of a “miss”. A node will be updated with $p_{m}^{\prime }$ if it is traversed by rays and corresponding endpoints are outside range $s_{c}$.
- $p_{\min}$ is the lower clamping threshold, which is the lower bound on the probability.
- $p_{u}$ is the upper bound on the probability derived by the average distance from a point to its k-NN.
- $p_{l}$ is the lower bound on the probability derived by the average distance from a point to its k-NN.
- *k* is the number of nearest neighbouring points.

### 3.2. Distribution of Average Distances

The average distance of a point is computed by searching its k-NN in the corresponding point cloud among points whose distances to the sensor are within range $s_{c}$. We use different distributions, i.e., Generalised Extreme Value (GEV) distribution, log-logistic distribution, Rayleigh distribution, Kernel Density Estimation (KDE) and normal distribution, to fit the average distances of all the points within range $s_{c}$ in a point cloud set generated from one data set. The results are presented in Section 4.4. The CDF of the average distance is nonsensitive to the types of distributions. Although KDE can fit the average distance better than other distributions, it would be difficult to change the k-NN model if KDE is applied due to its non-parametric property. Since there is no obvious change in CDF when the distribution is different, we assume that the average distance is subject to a normal distribution:

(8) $$f\left(x\right)=\frac{1}{\sqrt{2\pi \sigma^{2}}}\exp\left[-\frac{{\left(x-\mu \right)}^{2}}{2\sigma^{2}}\right]\;,$$

where μ is the mean and σ is the standard deviation.

Using the model in Section 3.1 to update occupancy maps, the mean and the standard deviation of the average distances are required. To avoid brute force calculation and improve accuracy, the method for calculating corrected sums of squares and products noted in [27] is implemented, which can reduce rounding errors in computer implementation. As a result, a series of values of mean and standard deviation will be generated as the number of points grows. Let *n* denote the number points. For i=1,2,⋯,n, the following process will be performed:

(9) $$\left\{ \begin{array}{cc} \mu_{i} & =\frac{i-1}{i}\mu_{i-1}+\frac{1}{i}s_{i}\\ Q_{i} & =Q_{i-1}+\frac{i-1}{i}{\left(s_{i}-\mu_{i-1}\right)}^{2}\\ & =Q_{i-1}+\left(s_{i}-\mu_{i-1}\right)\left(s_{i}-\mu_{i}\right) \end{array}\;,\right.$$

where $s_{i}$ is the average distance from the *i*th point to its k-NN in the corresponding point cloud, $\mu_{i-1}$ and $\mu_{i}$ are the mean values for i−1 and *i* points, $Q_{i-1}$ and $Q_{i}$ are the sums of the squares of the deviations for i−1 and *i* points, and $\mu_{0}=0$ and $Q_{0}=0$. Then, the mean and the standard deviation of the normal distribution can be derived by:

(10) $$\left\{ \begin{array}{c} \mu =\mu_{n}\\ \sigma =\sqrt{\frac{Q_{n}}{n}} \end{array}.\right.$$

### 3.3. Map Update

Algorithm 1 shows the mapping process with the k-NN-based inverse sensor model in Section 3.1. Here, $z_{t}$ represents the point cloud at time *t*. The corresponding position of the vision sensor is denoted as $x_{t}$. *m* is the occupancy map. Lines 3 through 10 update the nodes containing points. Lines 11 to 20 update the nodes traversed by rays cast from the sensor to endpoints. The traversed nodes are updated with log-odds values $l_{in}$ and $l_{out}$, corresponding to the probabilities of $p_{m}$ and $p_{m}^{\prime }$.
**Algorithm 1:** Map Update
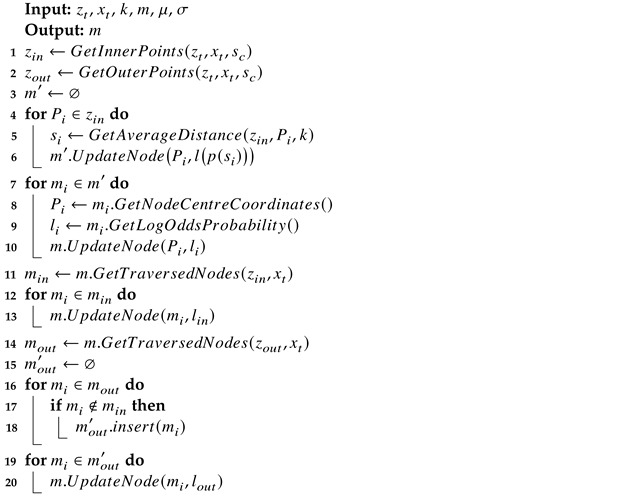


### 3.4. Parameter Space Considerations

In this work, we use grid parameter space for analysis, which has been introduced in [22]. Each parameter is generated by the corresponding maximum, minimum and step. A set, as introduced in [22], can be used to describe the possible values of any parameter:

(11) TTmax−TminTs|T=Tmin+(i−1)Ts,i<Tmax−TminTs+1,i∈N+,

where $T_{\max}$ and $T_{\min}$ are the upper and lower bounds on the parameter, and $T_{s}$ is the step.

The algorithm-required relations with other parameters should also be considered when generating parameters. $p_{m}^{\prime }\geq p_{m}$ since $p_{m}^{\prime }$ corresponds to points further from the sensor than $p_{m}$. A reasonable set of the parameters of the k-NN method should satisfy:

(12) $$\left\{ \begin{array}{c} 1>p_{\max}\geq 0.5>p_{m}^{\prime }\geq p_{m}\geq p_{\min}>0\\ p_{\max}\geq p_{t}\geq p_{\min}\\ p_{u}\geq p_{l} \end{array}\;.\right.$$

Two functions are defined in [22] to describe the combinations of parameters:

(13) $$g\left(T\right)=\left\lfloor \frac{\max(T)-\min(T)}{T_{s}}\right\rfloor +1$$

and

(14) h(T,T′,n)=minmin(T)+(n−1)Ts−min(T′)Ts′,g(T′)−1min(T)+(n−1)Ts−min(T′)Ts′+1,

where *T* and $T^{\prime }$ are the possible values of two parameters, $T_{s}$ and $T_{s}^{\prime }$ are corresponding steps, and *n* is an input combination number.

Considering the relations in (12), the number of combinations of the parameters in the k-NN model is:

(15) $$N_{k}=g\left(k\right)\left(\sum_{i=1}^{g(p_{u})}h\left(p_{u},p_{l},i\right)\right)\left(\sum_{i=1}^{g(p_{\max})}\sum_{j=1}^{g(p_{m}^{\prime })}\sum_{q=1}^{h(p_{m}^{\prime },p_{m},j)}\sum_{w=1}^{h(p_{m},p_{\min},q)}g\left(p_{t}\right)\right)\;,$$

where the possible values of $p_{t}$ correspond to the $p_{\max}$ value, $\min\left(p_{\max}\right)+\left(i-1\right)\tau \left(p_{\max}\right)$, and $p_{\min}$ value, $\min\left(p_{\min}\right)+\left(w-1\right)\tau \left(p_{\min}\right)$, and $\tau \left(T\right)=T_{s}$.

Our investigation of parameters is presented based on different data sets, an overview of which is given in Section 4.1. Let $N_{d}$ denote the number of data sets. We first generate a random permutation of the indices of all the possible combinations of k-NN parameters and then divide the combinations into $N_{d}$ groups according to their indices. The number of combinations for each data set is:

(16) $$N_{t}=\frac{N_{k}}{N_{d}}\;.$$

### 3.5. Parameter Reduction and Optimisation

We use the method in [22] for parameter reduction and optimisation, first identifying the most significant parameters and then optimising them with grid search.

Both point cloud parameters and mapping parameters will affect the mapping performance. However, given that mapping parameters have a higher impact on the mapping performance than point cloud parameters [22], in this work, we only focus on the reduction and optimisation of k-NN mapping parameters. We use 20 data sets introduced in [22] for experiments. Since the data sets in this work are collected in outdoor environments, most elements in an occupancy map belong to TNs. As introduced in [22], we use the Receiver Operating Characteristic (ROC) variant true positive rate (TPR)–false positive rate (FDR) as a performance metric to deal with such unbalanced data sets.

For parameter reduction, the combination of point cloud parameters remains the same on different data sets. We choose point cloud sets with FDR derived by the non-parametric naive mapping approach proposed in [22] based on their ranks. The choice of the point cloud set or the combination of point cloud parameters for k-NN parameter reduction will be given in Section 4.3. For each combination of k-NN parameters, an occupancy map can be generated using Algorithm 1. By comparing the map with ground truth, the nodes in the map can be classified into four categories, i.e., TPs, FPs, TNs and FNs. Metrics TPR and FDR are computed from the number of nodes in each category. NCA feature selection [23] will be applied to analyse parameter weights under the performance metrics TPR and FDR, which has been introduced in [22]. For ease of comparison, we normalise the weights derived by different data sets as per [23].

Based on the above parameter reduction and the optimisation results of OctoMap parameters in [22], parameters to be optimised can be determined and will be introduced in Section 4.3. Parameters can be optimised by grid search in the parameter space defined in Section 3.4. The area under the curve (AUC) of TPR–FDR variant is used as the performance metric for optimisation. Three point cloud sets in each data set will be selected using the naive approach in [22] to compare the best AUC derived by the k-NN method and that derived by OctoMap.

### 3.6. Run Time

For OctoMap, the time elapsed is proportional to the number of points processed, so the run time can be denoted as:

(17) t=aN+b,

where *a* and *b* are coefficients, and *N* is the number of points in the point cloud set used for generating an occupancy map.

The run time of the k-NN-based mapping approach is proportional to parameter *k* and the number of processed points *N*. The run time can be derived by:

(18) t=(ak+b)N+c,

where *a*, *b* and *c* are coefficients.

## 4. Experiments

### 4.1. Overview of Data Sets

**Figure 1:**
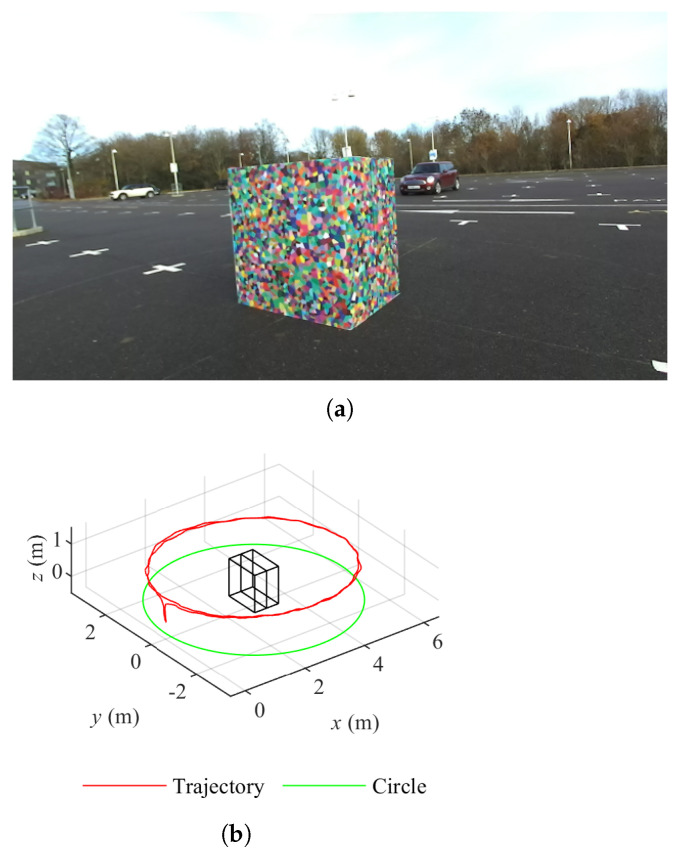
Overview of data sets. (**a**) O layout boxes of Voronoi diagrams in the parking lot. (**b**) Camera trajectory of O layout boxes of Voronoi diagrams in the parking lot.

We have introduced the data sets collected with a controlled procedure in [22]. Boxes with either a plain cardboard texture or Voronoi diagrams [28] are the targets to be explored. With a pair of boxes, five layouts can be created with the free tetrominoes in the Teris game [29,30], i.e., I, O, T, L and S. Data sets are collected in two environments, in front of buildings and in a parking lot, with a ZED stereo camera (Stereo Labs, USA) moving along the circle on the ground and orbiting the targets twice. The resolution of a single image is HD (1280 × 720 pixels). The number of data sets is 20 considering textures, layouts and environments. Figure 1a shows one example of 20 data sets, O tetromino layout boxes with the Voronoi pattern in the parking lot. The corresponding camera trajectory for data collection is presented in Figure 1b.

### 4.2. Map Generation and Node Classification

**Figure 2:**
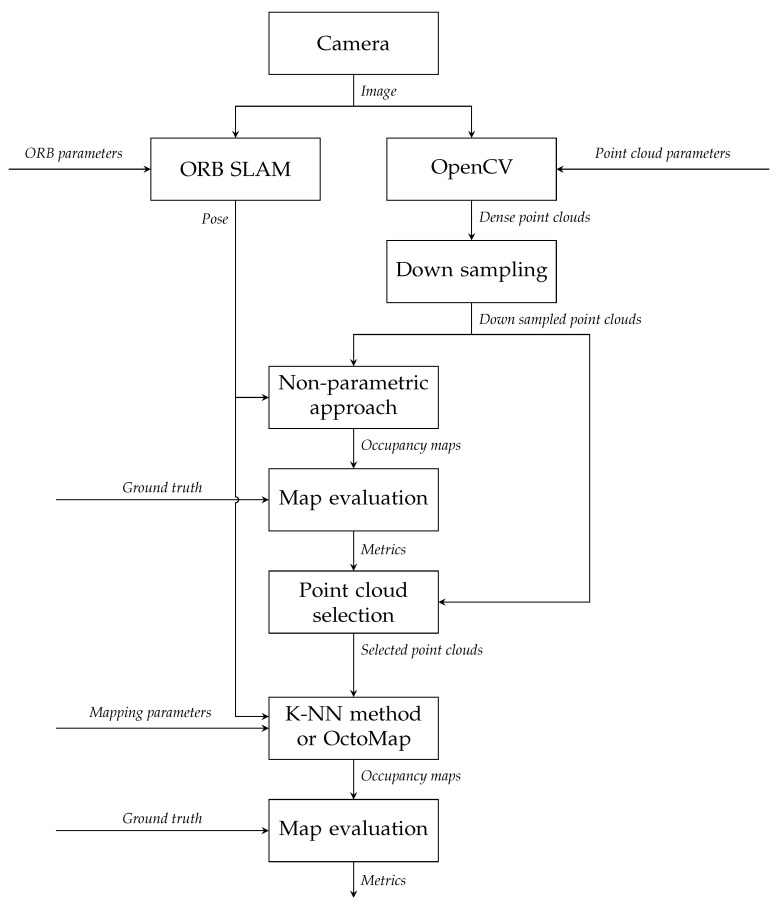
Design of experiments.

Figure 2 shows the experimental method for comparing two mapping methods. As explained in [22], the StereoSGBM algorithm [21] in OpenCV is implemented on the images of the keyframes derived by ORB-SLAM [31] to generate disparity maps, from which point clouds can be reconstructed with a stereo camera model. These point clouds are then downsampled with the Voxel filter in the PCL [7]. The resolution of the Voxel filter is set to 0.1 m. With time stamps, each point cloud can match with the keyframe pose generated by ORB-SLAM. The resolution of occupancy maps and maximum range $s_{c}$ for how long individual beams are inserted are set to 0.1 m and 4 m, which has been discussed in [22].

For k-NN parameter reduction, the combination of point cloud parameters remains the same on different data sets. Point cloud sets are chosen with FDR derived by the non-parametric mapping approach proposed in [22] based on their ranks. The choice of the point cloud set or the combination of point cloud parameters for k-NN parameter reduction will be given in Section 4.3. For each combination of k-NN parameters, an occupancy map can be generated from Algorithm 1. By comparing the map with ground truth, the nodes in the map can be classified into four categories using the method in [22]. Metrics TPR and FDR are computed from the number of nodes in each category. NCA feature selection proposed in [23] will be applied to analyse parameter weights, which has been introduced in [22]. For ease of comparison, the weights derived by different data sets are normalised as per [23].

Parameters can be optimised by grid search in the parameter space defined by the method in Section 4.3. Based on the above k-NN parameter reduction results and the optimisation results of OctoMap parameters in [22], parameters to be optimised can be determined and will be introduced in Section 4.3. The AUC of TPR–FDR variant is used as the performance metric for optimisation. Three point cloud sets in each data set will be selected using the non-parametric approach to compare the k-NN method with OctoMap.

In the end, the time estimation models introduced in Section 3.6 will be verified.

### 4.3. Parameter Space for Analysis

In [22], there are 1600 combinations of point cloud parameters, which means that 1600 point cloud sets can be generated for each data set. As specified in Section 3.5, we ranked the point cloud sets by FDR derived by the non-parametric mapping approach as per [22]. (1) For the reduction of k-NN parameters, we can fix the combination of point cloud parameters since they are less important than mapping parameters in performance [22]. We choose the combination of parameters corresponding to the 800th ranked point cloud set of the data set collected with I layout Voronoi boxes in front of buildings. (2) For the optimisation of the mapping parameters, we choose the 1st, 800th and 1600th (lower number indicates better quality, i.e., cleaner point clouds) ranked point cloud sets from each data set to compare the optimal performance of OctoMap and the k-NN method.

The configuration of mapping parameters is shown in Table 1. $p_{\max}$, $p_{m}$, $p_{t}$ and $p_{\min}$ are shared by the two mapping approaches. The choice of the step of the OctoMap parameters has been discussed in [22]; 0.12 is a reasonable step and will not affect the results. Smaller steps have been tested in [22] but no obvious difference has been observed in the results. The step is small enough for the grid search to give valid results. To investigate k-NN parameter weights, $p_{\max}$, $p_{\min}$ and *k* are varied with corresponding steps, and $p_{t}$ changes with $p_{\max}$ and $p_{\min}$. With (15), the total number of combinations is 112,500. To optimise the k-NN parameters, based on the results in Section 4.4, *k* is constant since it has a lower impact on the performance metrics. $p_{\max}$ and $p_{\min}$ are set to 0.98 and 0.02 since these values show the highest frequencies in the optimal values in [22]. Moreover, $p_{\max}$ and $p_{\min}$ are the upper and lower bounds on the probability but not the parameters for inverse senor models, and they are shared by both the k-NN method and OctoMap. Therefore, locking these two parameters does not benefit any approach but can decrease the number of combinations of parameters to reduce the computational time. Besides $p_{\max}$ and $p_{\min}$, the setup of other OctoMap parameters is dependent on the configuration in [22]. To compare the k-NN method and OctoMap, the combinations of parameters for the two mapping algorithms are 4050 and 180, respectively.

### 4.4. Results

**Figure 3:**
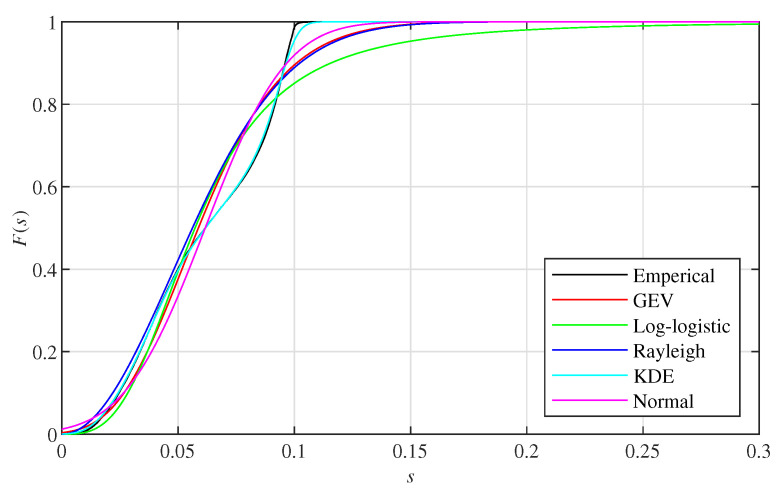
Cumulative Density Function (CDF) of the average distance fitted by different distributions.

We first show the results of the average distance fitted by different distributions, i.e., GEV distribution, log-logistic distribution, Rayleigh distribution, KDE and normal distribution. An example is given by the average distance derived by the 800th point cloud set of I layout Voronoi boxes in front of buildings. The corresponding CDF is presented in Figure 3. Results show that the CDF of the average distance is nonsensitive to the types of distribution. Results from other point cloud sets also show a similar conclusion.

**Figure 4:**
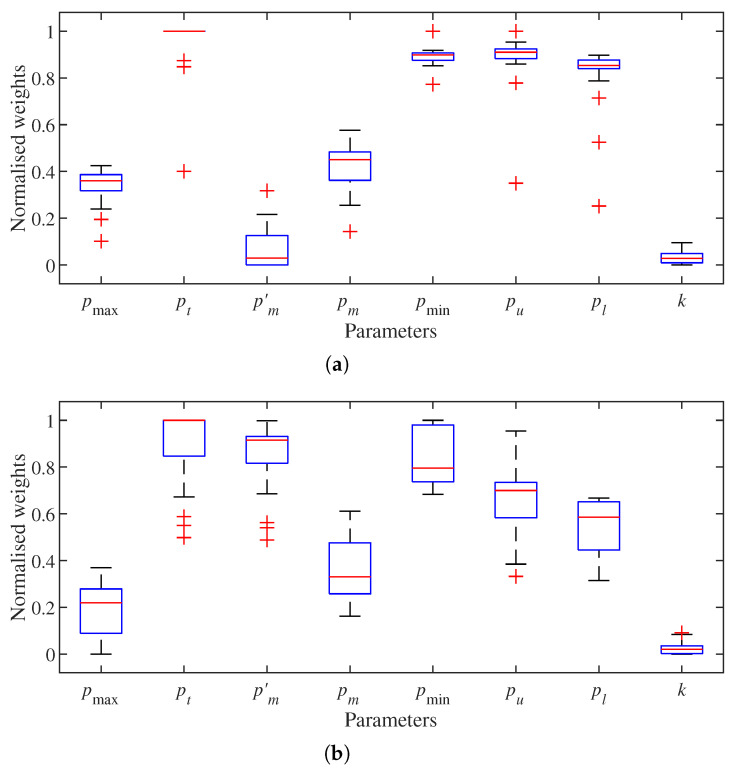
Normalised weights of k–Nearest Neighbours (k–NN) parameters on different performance metrics. (**a**) True positive rate (TPR). (**b**) False discovery rate (FDR).

Then, the results of the weights of k-NN parameters under performance metrics TPR and FDR are presented. As specified in Section 4.3, point cloud parameters are consistent in the 20 data sets. With the configuration of mapping parameters in Table 1, the weights of k-NN parameters are computed by implementing NCA feature selection on TPR and FDR derived by the node classification results. With (15) and (16), the number of parameter combinations for each data set is 5625. The normalised weight of each k-NN parameter is shown in Figure 4. Overall, parameter *k* is less important than the other parameters. Its weight is under 0.1 in both performance metrics. Therefore, parameter *k* can be excluded from the optimisation for the best mapping performance.

**Figure 5:**
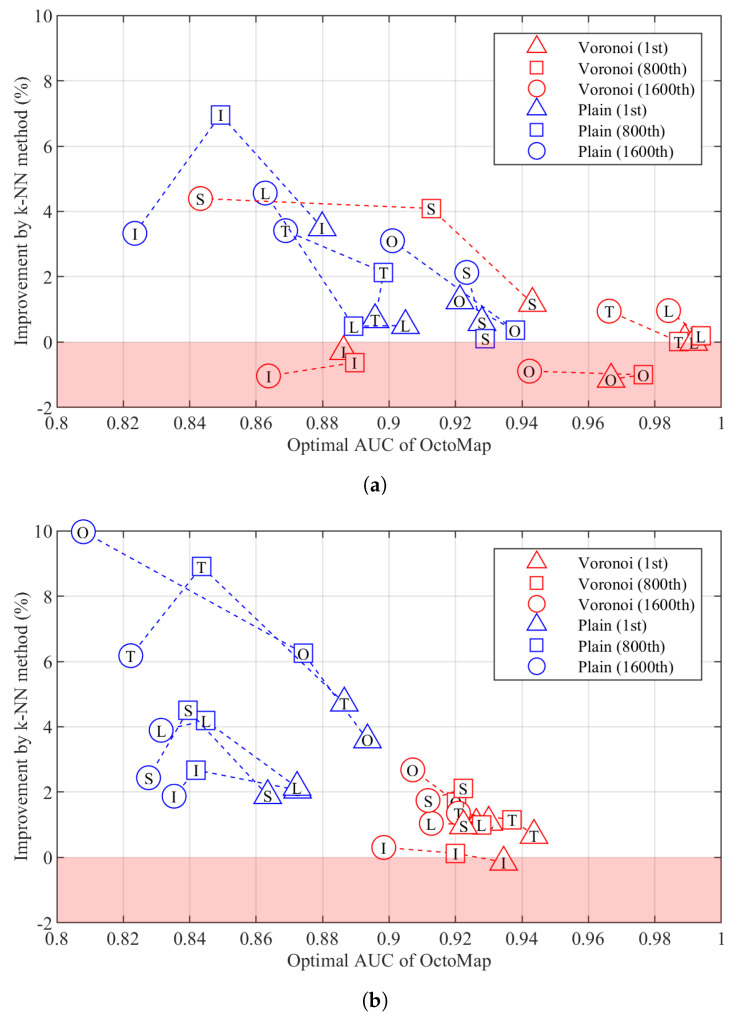
Improvement by the k–Nearest Neighbours (k–NN) method over the optimal area under the curve (AUC) of OctoMap. (**a**) Building. (**b**) Parking lot.

We also compare the performance of the k-NN method and OctoMap. The 1st, 800th and 1600th ranked point cloud sets generated from each data set are selected to optimise the performance of each mapping algorithm. The optimal performance of the two mapping algorithms is then compared. All the parameters except $p_{\max}$, $p_{\min}$, $p_{t}$ and *k* will be optimised by searching the optimal AUC of TPR–FDR variant using the grid parameter space defined with Table 1. $p_{t}$ is varied to generate points on the TPR–FDR curve. For any combination of parameters in each mapping algorithm, nine points will be produced. A point will be discarded if its metric is not a number. Figure 5 shows the improvement achieved by the k-NN method over the optimal AUC derived by OctoMap on 20 data sets. The improvement increases as the optimal AUC of OctoMap decreases, but can be negative when the AUC of OctoMap is relatively large. With the parameter configuration in Table 1, we can achieve an improvement up to 10%. Overall, the mapping performance of Voronoi boxes is better than that of plain boxes.

**Figure 6:**
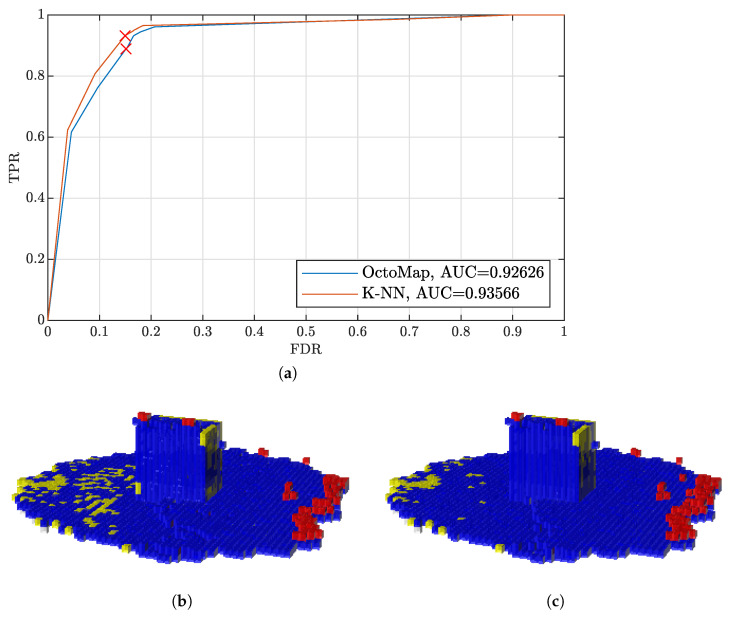
Occupancy maps derived by different algorithms using the data set of O layout Voronoi boxes in the parking lot. (**a**) Receiver Operating Characteristic (ROC) variant true positive rate (TPR)–false positive rate (FDR). (**b**) Occupancy map derived by OctoMap. Blue: TPs, red: FPs and yellow: FNs. TNs are not included for clarity. (**c**) Occupancy map derived by the k–Nearest Neighbours (k–NN) method.

Figure 6 shows an example of the occupancy maps derived by OctoMap and the k-NN method. The TPR–FDR curves of the two mapping algorithms are presented in Figure 6a, corresponding to the improvement using the first point cloud set of the data set of the O tetromino layout of Voronoi boxes in the parking lot in Figure 5. We pick up two points of similar FDR on the two curves, and corresponding maps are shown in Figure 6b,c.

**Figure 7:**
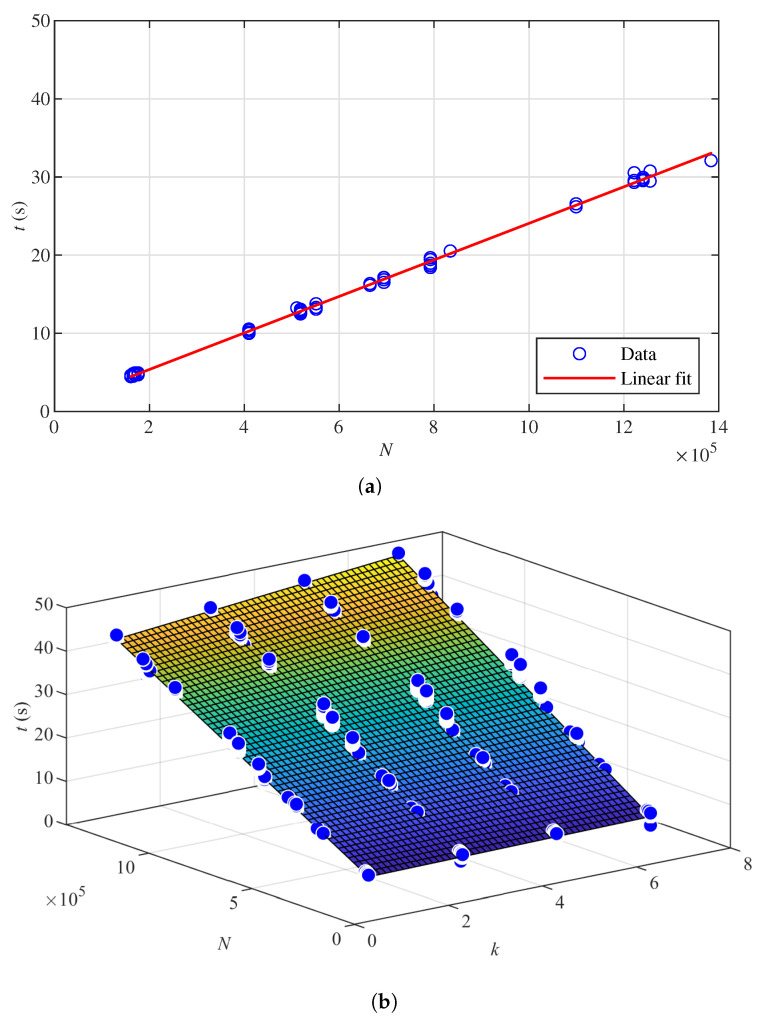
Run time. (**a**) Linear regression for the run time of OctoMap. (**b**) Polynomial regression for the run time of the k–Nearest Neighbours (k–NN) method.

Finally, the time model in Section 3.6 is verified. Here, the point cloud parameters are the same as those used for k-NN parameter reduction, introduced in Section 4.3. Figure 7a presents the linear fit for the run time of OctoMap. Corresponding coefficients in (17) are $a=2.3371\times 10^{-5}$ and $b=6.9527\times 10^{-1}$. Then, 5% of the parameter reduction results with the aforesaid point cloud parameters in [22] are randomly selected to plot Figure 7a. The result shows that the run time is proportional to the number of points processed by the OctoMap algorithm. Figure 7b verifies the time estimation model (18). In each data set, 5% of the k-NN parameter reduction results are randomly selected to estimate the time model. The coefficients in the model are $a=7.3592\times 10^{-7}$, $b=2.7681\times 10^{-5}$ and c=1.5148. The result shows that run time increases with parameter *k* and the number of points processed.

### 4.5. Discussion

The CDF of the average distance is nonsensitive to different distributions. Given that the CDF is implemented to define the relationship between the average distance and the change in the occupancy probability, we use a parametric distribution for ease of adjusting the k-NN model. This can be potentially useful since the distribution of the average distance might be different when the environment is changed. With a parametric distribution, the k-NN model can be changed to adapt to different environments.

Among all the k-NN parameters, parameter *k* has a lower impact on the performance metrics and thus can be fixed. In addition, based on the optimisation of OctoMap parameters in [22], $p_{\max}$ and $p_{\min}$ can be set as constants to further reduce the computational complexity.

The optimal AUC of k-NN shows an improvement of up to 10% over that of OctoMap. The improvement achieved by the k-NN method increases as the optimal AUC of OctoMap decreases but can be negative when the optimal AUC of OctoMap is relatively large. Overall, the mapping performance is better in the environment with buildings since there are more image features on the objects nearby and the quality of point clouds is better. In each environment, the mapping performance is normally better when targets are covered with Voronoi diagrams due to the extra features introduced by the diagrams. There is no obvious trend among different layouts.

In Figure 5, the improvement by the k-NN method against OctoMap can be observed on most data sets in terms of AUC. However, when the k-NN method achieves optimal improvement, the TPR–FDR curves derived by the two mapping algorithms may intersect, i.e., points on the curve derived by the k-NN method are always better than those on the curve derived by OctoMap when FDR is smaller than that of the intersection point, while they are worse when FDR is larger than that of the intersection point. However, normally, a combination of k-NN parameters can be found whose improvement against the optimal AUC of OctoMap is less significant than that achieved in Figure 5 such that, for each point on the TPR–FDR curve of OctoMap, a point of better performance can be found on the curve derived by the k-NN method.

For both occupancy mapping methods, run time is proportional to the number of points processed. OctoMap is faster than the k-NN method when processing the same point cloud set. The run time of the k-NN method increases with parameter *k*. Since the change in *k* has little impact on the performance metrics, a smaller *k* can be chosen to reduce the computational time.

## 5. Conclusions

In this paper, we present an inverse sensor model for occupancy mapping using the context of neighbouring points. The occupancy information of a point is represented with the average distance to its k-NN. The relationship between the average distance and the occupancy probability is defined with the corresponding CDF. By implementing NCA, the parameter that has a lower impact on the mapping performance can be reduced. In addition, by considering the optimal values of OctoMap parameters, the number of parameters to be investigated can be further reduced. Through searching the grid parameter space, the residual most important parameters can be optimised to achieve the optimal performance. We implement the k-NN method on point clouds derived by different data sets. Results show that the k-NN method is effective in improving performance over OctoMap. Through our analysis, the key findings are as follows:

- The k-NN model is nonsensitive to different types of distributions.
- Parameter *k* is of lower impact than other k-NN parameters.
- Through grid search optimisation, the optimal performance of OctoMap can be improved by the k-NN method.

In the future, the computational time can be optimised. The number of combinations of parameters can be potentially reduced before computation. Point clouds generated from other types of sensors will be used to test our method. In addition, the distribution of the average distance in different environments can be investigated.

## Figures and Tables

**Table 1:** Configuration of mapping parameters.

Parameter	Minimum	Maximum	Step	Method
$p_{\max}$ ^a^	0.5	0.98	0.12	k-NN
$p_{\max}$ ^b^	0.98	0.98	N/A	Both
$p_{h}$	0.5	0.98	0.12	OctoMap
$p_{m}$	0.02	0.38	0.12	Both
$p_{m}^{\prime }$	0.02	0.38	0.12	k-NN
$p_{\min}$ ^a^	0.02	0.38	0.12	k-NN
$p_{\min}$ ^b^	0.02	0.02	N/A	Both
$p_{t}$	$p_{\min}$	$p_{\max}$	0.12	Both
$p_{u}$	0.02	0.98	0.12	k-NN
$p_{l}$	0.02	0.98	0.12	k-NN
*k* ^a^	1	7	2	k-NN
*k* ^b^	1	1	N/A	k-NN

^a^ Configuration for the reduction of k-NN parameters. ^b^ Configuration for the optimisation of OctoMap parameters and k-NN parameters.

## Data Availability

Data sets are available at https://doi.org/10.15125/BATH-00594 (accessed on 15 November 2021) under the Creative Commons Attribution 4.0 license. The software implementation of the k-NN method is available at https://github.com/dlmiaoyu/KNNMapping (accessed on 15 November 2021).
